# Unveiling family relationships based on the context of domestic violence: a Grounded Theory^*^

**DOI:** 10.1590/1980-220X-REEUSP-2023-0009en

**Published:** 2023-12-22

**Authors:** Vanessa Carla Batista, Mayckel da Silva Barreto, Nadirlene Pereira Gomes, Eleandro Prado, Stela Maris de Mello Padoin, Fernando José de Godoy, Tatiane Herreira Trigueiro, Sonia Silva Marcon

**Affiliations:** 1 Universidade Estadual de Maringá, Programa de Pós-Graduação em Enfermagem, Maringá, PR, Brazil.; 2 Universidade Federal da Bahia, Escola de Enfermagem, Salvador, BA, Brazil.; 3 Universidade do Vale do Itajai, Biguaçu, SC, Brazil.; 4 Universidade Federal de Santa Maria, Programa de Pós-graduação em Enfermagem, Santa Maria, RS, Brazil.; 5 Secretaria Municipal de Saúde de Umuarama. Unidade de Pronto Atendimento, Umuarama. PR, Brazil.

**Keywords:** Domestic Violence, Grounded Theory, Family Relations, Nursing Care, Women′s Health, Violencia Doméstica, Teoría Fundamentada, Relaciones Familiares, Atención de Enfermería, Salud de la Mujer, Violência doméstica, Teoria Fundamentada, Relações Familiares, Cuidado de Enfermagem, Saúde da Mulher

## Abstract

**Objective:**

To understand the meanings attributed to family relationships by women who have experienced domestic violence.

**Method:**

Explanatory study using Symbolic Interactionism and Grounded Theory as references. Data were collected from March to November 2021, through online interviews with 23 women found on the social media application *Facebook*®.

**Results:**

Data analysis allowed the construction of a theoretical model consisting of three processes: “We learned that it was normal to be mistreated”: experiencing a context of violence in the family of origin; “I just wanted a family”: experiencing partner violence and redefining family relationships in the context of violence.

**Conclusion:**

The meanings attributed to family relationships are elaborated and modified according to the interpretation, trajectory of confrontation, and interactions of women with other individuals and objects in the social web, over time. When experiencing domestic violence, women give new meaning to their feelings and begin to attribute new values, emotions, and empathy to family relationships, expanding their understanding of their weaknesses and potential.

## RESUMO

**Objetivo:**

Compreender os significados atribuídos às relações familiares, por mulheres que vivenciaram violência doméstica.

**Método:**

Estudo explicativo que utilizou como referenciais o Interacionismo Simbólico e a Teoria Fundamentada nos Dados. Os dados foram coletados no período de março a novembro de 2021, por meio de entrevistas on-line com 23 mulheres localizadas na rede social *Facebook*®.

**Resultados:**

A análise dos dados permitiu a construção de um modelo teórico, constituído por três processos: “A gente aprendeu que era normal ser maltratada”: experienciando um contexto de violência na família de origem; “Eu só queria uma família”: vivenciando violência pelo companheiro e Ressignificando as relações familiares no contexto de violência.

**Conclusão:**

Os significados atribuídos às relações familiares são elaborados e modificados conforme a interpretação, trajetória no enfrentamento e interações das mulheres com outros indivíduos e objetos no tecido social, ao longo do tempo. Ao vivenciar a violência doméstica as mulheres ressignificam seus sentimentos e passam a atribuir novos valores, emoções e empatia às relações familiares, ampliando a compreensão de suas fragilidades e potencialidades.

## RESUMEN

**Objetivo:**

Comprender los significados atribuidos a las relaciones familiares por mujeres que han vivido violencia doméstica.

**Método:**

Estudio explicativo que utilizó como referentes el Interaccionismo Simbólico y la Teoría Fundamentada. Los datos fueron recolectados de marzo a noviembre de 2021, a través de entrevistas online a 23 mujeres encontradas en la red social *Facebook* ^®^.

**Resultados:**

El análisis de los datos permitió la construcción de un modelo teórico, compuesto por tres procesos: “Aprendimos que era normal que nos maltrataran”: experiencia en un contexto de violencia en la familia de origen; “Solo quería una familia”: experiencia de la violencia en la pareja y redefinición de las relaciones familiares en el contexto de la violencia.

**Conclusión:**

Los significados atribuidos a las relaciones familiares se elaboran y modifican según la interpretación, trayectoria de confrontación e interacciones de las mujeres con otros individuos y objetos del tejido social, a lo largo del tiempo. Al vivir violencia doméstica, las mujeres dan un nuevo significado a sus sentimientos y comienzan a atribuir nuevos valores, emociones y empatía a las relaciones familiares, ampliando su comprensión de sus debilidades y potencialidades.

## INTRODUCTION

Domestic violence (DV) remains the most common event of violence against women and represents a major threat to public health. The harmful consequences not only involve women, but also put at risk the entire family system and its organization, which is indispensable for its proper development and functionality^(1)^.

Globally, one in three women has experienced physical and/or sexual violence from a partner or sexual violence from any perpetrator in her life^(2)^. Regarding feminicide as a result of DV, in 2019, in Brazil, around 3,737 women were killed. These data indicate Brazil as one of the countries with the most violent rates of this type of violence in the world^(3,4)^.

Statistically, DV takes place mainly in the home environment, considered by many a space of refuge and protection, but which, in these cases, facilitates the practice and concealment of violence^(5)^. Furthermore, DV is influenced by different aspects – sociocultural, psychological, behavioral and economic; causes family instability; impacts on the morbidity and mortality of those involved; violates human rights; and is often accompanied by silence and submission of women in situations of violence^(6)^.

Generally and historically, women’s submission is linked to the social roles attributed to men and women, which are the results of a process of social naturalization of biological, gender, and cultural transformation differences that convert into male domination and subtly result in violence that is incorporated into common life. Thus, it is possible to understand that DV may have been trivialized, as the dominance of men over women was naturalized in socio-affective, work, and power relationships, as well as in the way people construct and perceive life and the world surrounding them^(7)^.

In this regard, authors who seek to understand the complexities that permeate the phenomenon of DV have highlighted an intergenerational character that involves both the woman and the aggressor, characterized by the historical reproduction of violence, which begins in childhood and/or adolescence and continues throughout adulthood. Such repeated reproduction occurs based on learned behaviors and values, which are naturalized among different social groups, mainly the family^(1)^.

Family relationships and the violent context, when linked, can generate negative consequences that go beyond affective relationships, extending to social, personal, and psychological spheres, as they legitimize violence as a conflict resolution strategy in the most diverse situations^(8)^. Thus, the path to be taken in the search to interrupt the cycle of DV is multifaceted and requires greater understanding of the complexity involved in inserting and maintaining women in this situation.

Observing the lack of investigations addressing the specific context of DV, from the perspective of women, linked to family relationships, we identify the need for explanatory studies allowing the expansion of the understanding of this phenomenon, an in-depth investigation on social knowledge of DV for guidance of care practices at different levels and forms. Based on this assumption, the objective of the present study was to understand the meanings attributed to family relationships by women who experienced DV.

## METHOD

### Design of Study

This is an explanatory study using Symbolic Interactionism (SI) and Grounded Theory (GT) as technical framework. SI is a theoretical perspective centered on human interaction, according to which human beings act as per the meanings they attribute to things^(9)^. GT, which has conceptual bases in SI, aims to allow the construction of a theoretical model that facilitates the understanding of social phenomena, from the perspective of the subjects investigated^(10)^. In this study, the steps recommended by the Constructivist aspect of GT were followed^(11)^. To write the study report, criteria established in the *Consolidated criteria for reporting qualitative research* (COREQ)^(12)^ were used as a support tool.

### Population, Local, and Selection Criteria

The informants were women from different regions of Brazil who were part of support groups for people in situations of violence, hosted on the social media application *Facebook®.* These are private groups, that is, the inclusion/admission of new participants is evaluated by their administrators. Therefore, the researcher requested participation in six groups, randomly selected, among those whose title explained the issue of domestic violence. At the beginning of her participation in the groups, the researcher sought to establish some form of bond with the others, expressing personal and/or professional support for reports that revealed anguish, fears, and the search for help. After a month in the groups, the researcher requested authorization from the administrators to insert a brief invitation for participation in the study. There were around eight comments on the invitation post in each group, which were responded to, with private contact being established so that the researcher could better clarify the objective of the study and the type of participation desired.

Women who consented to participate in the study were provided with telephone contact to carry out the interview on a day and time of their preference, moment when they were informed about the Free and Informed Consent Form (FICF) and the inclusion and exclusion conditions were assessed. Inclusion criteria were being a woman, being 18 years old or over, having experienced DV by a former partner, and having access to the internet. The exclusion criterion was the cancellation of the appointment or failure to appear for an interview in the online environment after three attempts.

### Data Collection

Data were collected from March to November 2021, through open interviews carried out in a virtual environment. Considering the assumptions of GT, data collection occurred simultaneously with the transcription and analysis of interviews with 23 participants distributed into four sample groups. The first group was intentionally made up of seven women who experienced different forms of violence. At that time, the purpose was to comprehensively explore the relationships established in the violent context of the house.

During the interview collection and analysis process, it was evident that the repercussions of DV were influenced by different factors, including the presence or absence of children. All participants in the first sample group did not have children with the perpetrator of violence. This context triggered the following questions: Would the aggressor behave differently if he had children with the woman or even if the child(ren) they already had were his? These, in turn, led to the formulation of the following hypothesis: The woman’s intention to break the cycle of violence is modulated by the aggressor’s behavior and, therefore, is different when she has children with him.

Thus, the second sample group consisted of six women who had children with the perpetrator of violence and were selected intentionally, as the selection was made among those who responded to an invitation published in the six support groups, in which the desire to contact women who had children with the aggressor was specified. Data obtained in this group showed that children played an essential role in the way women experienced violence, and even acted as a driving force in the search for changing the reality they lived. Based on this understanding, it seemed opportune to know the perspective of the children of women who experienced DV, leading to the third sample group, which consisted of three daughters, two of whom were daughters of the participants in the second sample group and one, the daughter of a victim of feminicide, who was known when the first sample group was formed. Through the data obtained in the third sample group, it was observed that, because they remain exposed to DV situations on a daily basis, the daughters carried scars as intense from this experience as their mothers, which influenced them in adult life and in affective relationships, making it necessary to investigate the experience of women with a history of violence in their family of origin. Thus, the fourth and final sample group was made up of seven women who, in addition to experiencing DV in adulthood, had a history of DV in childhood. To this end, once again a specific invitation was published in the six groups and the women who responded to this invitation and who showed interest in speaking freely about the topic were included in the study.

The interviews began with the guiding question *How do you understand your family relationships in the context of experienced violence?* and followed a pre-established script with open questions, allowing the incorporation of new questions from the outline of the answers. The interviews lasted an average of 60 minutes and were carried out solely through video calls, both on the social media application *Facebook* ^®^ and in *Whatsapp* ^®^ *,* according to the participant’s preference. All interviews were audio-recorded, carried out in a private location in the woman’s home and carried out by the main researcher of this study, who is a woman, white, with no personal history of DV, nurse, with doctorate degree in nursing, experienced in qualitative data collection, and who had no previous bond with the participants.

### Data Analysis and Treatment

The analysis followed the coding steps proposed by the Constructivist aspect of GT, namely: initial and focused coding. In the initial coding, data was fragmented and analyzed with the aim of conceptualizing ideas and/or meanings expressed by the participants, transforming them into codes. In the second stage, focused coding, the most significant codes were used that allowed better analytical understanding of the data. As certain concepts emerged with greater frequency and prominence, subcategories and categories were constructed, which were subsequently interconnected in an explanatory way, to allow the identification of the central phenomenon of the research^(10,11)^. It should be noted that memos and diagrams were used throughout the investigation process^(11)^. Data were interpreted based on Symbolic Interactionism.

### Ethical Aspects

Study developed in accordance with the guidelines of Resolution 466/12 of the National Health Council and in accordance with the guidelines for research procedures in a virtual environment of the National Research Ethics Commission (CONEP). Project approved by the Permanent Human Research Ethics Committee of the signatory institution (Opinion nº 4.426.287/2020). Participants’ consent was obtained by signing the Free and Informed Consent Form via *Google Forms*. To preserve participants’ anonymity, fictitious names of women who were significant to the main researcher were used, followed by the age and sample group to which they belonged (e.g.: *Wanderleia, 39 years old, GA2*)*.*

## RESULTS

Thirty-three women aged between 21 and 61 years participated in the study, 13 of whom Catholic, nine evangelical, and one Kardecist. The average income varied between one and three minimum wages and, regarding education, six had primary education, six had higher education (two of which were still studying), nine had studied up to high school, and two of them knew how to read and write. Regarding marital status, none of them were in a relationship with the author of the DV, with three being in a new relationship.

Data analysis allowed the construction of a theoretical model, whose central phenomenon was named: “**Giving meaning to family relationships in the context of domestic violence”,** consisting of three interconnected processes: *Process 1- “We learned that it was normal to be mistreated”: Experiencing a context of DV in the family of origin”,* in which the woman experiences, at an early stage, DV during childhood, together with other family members. In this context, violence is often carried out by the father figure, and the child gradually begins to introject meanings of tolerance to abusive relationships and the naturalization of violent relationships. *Process 2 - “I just wanted a family”: experiencing violence from a partner,* in which the woman perceives herself reproducing the violence experienced in her family of origin. This is sometimes reflected in experiencing marital relationships in a subdued way. When women find themselves in a new context of violence practiced by their partner against them and their children, they seek support in different social relationships, in a paradoxical way, mainly within the family system, with not all of them getting the expected support, experiencing a new abandonment process. Finally, *Process 3 - Reframing family relationships in the context of DV,* which illustrates that, when leaving the violent context, women can give new meaning to family roles, communication, and a sense of support, being welcomed within the family system and understanding family relationships and the violence experienced in childhood.

**Figure 1:**
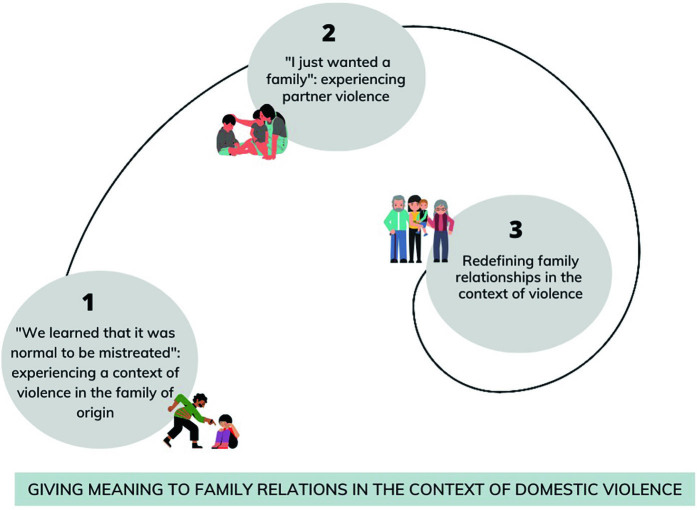
Representative diagram of the central Phenomenon: “Giving meaning to family relationships from the context of domestic violence.” (author, 2023).

The phenomenon identified in this study presents a non-linear sequence of events, as women seem to live in an eternal loop of violent events, as they begin life with DV and later find themselves in violent marital relationships, which often also affect their children. Furthermore, even if they manage to leave the violent context, the memories experienced on a daily basis return and continue, in a loop, present in these women. Consequently, each woman in a situation of DV represents a universe and situations of violence are part of a pattern that repeats itself from childhood to adulthood and remains in memories (Figure 1).

Process description *“We learned that it was normal to be mistreated”: Experiencing a context of violence in the family of origin”* expresses reflections on the past, raising nostalgic and sad memories, recovering memories of a childhood marked by scars resulting from witnessing, on a daily basis, their father’s violence against their mother, siblings, and themselves. According to the interviews, the father exercised control over the family mainly through physical force and psychological abuse, restricting freedom and rights, as the following statements demonstrate:

*In terms of love, affection, tenderness, I never had that from him* (father). *My sisters got married early to leave. I grew up seeing him hit my mother a lot, I remember that she always had a bruised eye because he hit her and us a lot. He always said that I was incapable of anything and that I would always depend on him*. (Aline, 23 years old, GA4)

*I have always lived with violence at home. My father beat us a lot, and we grew up in that environment. I couldn’t open the refrigerator, I couldn’t use the bathroom inside the house, because my father was disgusted by us and we were very scared*. (Maria, 36 years old, GA4)

It is clear that mothers remained silent and accepted violence as a way of minimizing their children’s exposure and due to the fear of not being able, alone, to provide the necessary subsistence for the family.

*My mother was an extremely submissive person and dependent on my father. I remember that when he hit her a lot, even with blood, she said: “Bad with him, worse without him.” I heard that phrase my entire childhood*. (...) *Whenever he came home drunk, he would also beat us, and besides, I had to watch my mother being beaten and stay quiet, because if I cried, he would beat us too. He shouted that he was the one in charge of our lives, and that was why he treated us the way he wanted*. (Patricia, 44 years old, GA4)

*My mother suffered domestic violence, and I witnessed everything. My father was very aggressive. We were afraid of him, so I also had a childhood experiencing all of this. My mother suffered in silence her entire life for more than 30 years, she was so silent that she became depressed... My mother was very unhappy*. (Wanderleia, 39 years old, GA2)

Data point to alcohol and other drugs as predominant elements in the violent family context. Remembering episodes in which the abusive use of these substances was associated with violence within the family triggered feelings of sadness in women.

*My father was an alcoholic, you know. My mother worked as a maid, supporting the house alone, because he spent the money on drinking and women*. (Maria, 36 years old, GA4)

*My father drank a lot, and when he got home he broke everything, threw the food away, and beat my mother a lot*. (Gabrielle, 32 years old, GA1)

The hostile context experienced from a very early age by women was responsible for the construction of meanings, especially related to insecurity, fear, and the feeling of constant vulnerability. This situation results from the violent environment to which they were subjected and the various situations of ignored abuse, especially by those who should protect them.

*My father had a friend and only today do I understand that he had other intentions, he was always touching my hair. I told my father several times and he was never bothered*. (Aline, 23 years old, GA4)

*I went through sexual abuse by an uncle and had no support from my grandmother. The feeling I had was that they wanted to suppress it as if it were normal, something that women have to accept. I have “flashes” of him putting* (his genitals) *in my mouth and me vomiting, I was very small* (...). *How come no one saw it?* (crying and pause)*. I’m angry about this story and how my family dealt with it, I think children have to be protected*. (Mariana, 42 years old, GA4)

Faced with complex experiences, flooded by feelings of lack of protection, fear, rejection, abandonment and indifference, there seems to be an imbalance and sometimes absence of feelings between the relationships established with the maternal figure from whom women expect, above all, reciprocation of affection.

*I was always very angry with my mother, because I saw her as responsible for the bad things that happened to me, I always had a complex of having been abandoned. She said things like, “you came here to destroy my life”* (...) *Before I got married, I remember she was sitting on the sofa and I put my hand on her head, she took my hand away and said: “you’re distressing me” Minutes later, my sister arrived and put her head in her lap* (long pause and crying) *and my mother started to caress her. That hurt me a lot. I felt like I didn’t belong there*. (Mariana, 42 years old, GA4)

*My mother never wanted to have a daughter, she never liked it and she made that very clear. She mistreated me a lot, she didn’t give me love or affection. I discovered what it was like to hug a woman as an adult*. (Elenice, 54 years old, GA1)

The different forms and expressions of violence suffered by women since childhood reverberate in the naturalization of abusive relationships in other contexts, in such a way that there seems to be a symbiosis between different and antagonistic feelings in the same relationship. That is, suffering becomes an important marker of the love received in their relationships, in such a way that women are led to believe that they can only have access to love when accompanied by pain and suffering.

*In my house, we learned that it was normal to be mistreated, this whole situation of abuse, we had to endure in silence*. (Maria, 36 years old, GA4)

*My mind created that to feel someone’s love, I have to feel pain, I didn’t know anything else, because that’s what I was used to receiving*. (Fátima, 32 years old, GA4)

Looking for ways to solve the problems experienced in the family of origin, some women reported that the solution was to devise strategies to escape the violent family context. When this opportunity appeared through new relationships, it was immediately embraced by them, in an attempt to experience what seemed to mean, in a way, a solution to what they experienced in their family of origin.

*I couldn’t wait to get out of that environment. I ran away from home, went to live with him* (the attacker) *and it was worse than if I had stayed at my father’s house. He realized that I had no one for me, that I was completely dependent on him, financially and emotionally*. (Patricia, 44 years old, GA4)

*I didn’t feel like I was part of that family, that place. Suddenly I meet a guy, and he seemed to be the solution to my problems. He came very friendly: “I’m going to help you, we’re going to get out of here, get married, you don’t have to go through this.” I saw my salvation in him*. (Mariana, 42 years old, GA4)

The way women interpret the family dynamics in which they were inserted in childhood, in which power was attributed to the paternal figure, was decisive in the formation of the lenses through which they observe and interpret the world. In view of the above, the meanings elaborated about their family relationships in the context of DV were, little by little, being constructed based on the different experiences.

### “I Just Wanted a Family”: Experiencing Violence from a Partner

Although they sought to form a family with distinct and opposite characteristics from those learned throughout childhood, women found themselves in a new context of violence that sometimes means a more harmful condition than that experienced in the family of origin. This is because, for many, this family was dreamed of and idealized together with their companions, who, when practicing DV, generated, in addition to all the suffering, the feeling of disappointment and frustration at finding themselves again in a context of violence.

*I was deeply disappointed. I don’t even know how to define it in words. I always asked myself why I had to live this if what I wanted was so simple, I just wanted a family*. (Mariana, 42 years old, GA4)

*It really wasn’t at all what I thought. That fantasy I created of building a family, being happy, was all an illusion*. (Gabrielle, 32 years old, GA1)

*We want to build a family, but at what cost? It could have cost me my life*. (Maria, 36 years old, GA4)

When they find themselves in a new context of violence, now occupying the role they previously witnessed, many of these women begin to face a new process of abandonment. In most cases, the first choice when they sought help was their family; however, once again, they are faced with a long and difficult path that will be walked alone.

*Once I called my mother and asked for help. She said: “daughter, I can’t do anything, your father doesn’t like you.” I felt like …, despicable. Right away, I hung up the phone, I didn’t have any tears to cry* (sigh). *I stood still, trying to get what she had told me.* (Patricia, 44 years old, GA4)

*When I said I was going to get separated, my father told me: “Bad with him, worse without him. At least with him you won’t lack food.”* (Wanderleia, 39 years old, GA2)

Based on these experiences, some women needed to understand the complexity of the phenomenon as it presented itself, seeking new ways and alternatives to face it and, often, in a solitary way.

*Until I understood that the decision to get out of that situation was mine and not someone else’s, that it would need to come from me, it took a long time*. (Maria, 36 years old, GA4)

*We don’t get support from the family within the relationship. Either you go against it and accept the family’s support or you stay against the family and hold back the wave of not being able to leave. There’s no way, it’s an emotional prison and the family can’t help*. (Vanuza, 47 years old, GA1)

The path taken by women, from the time they found themselves inserted in the context of DV until they finally managed to leave it, was long and doomed to continuous and lonely suffering. When experiencing the feelings learned on this path, new meanings were constructed and attributed to the objects that appeared in their realities, including family relationships.

### Reframing Family Relationships in the Context of DV

Insertion into a new context of violence raises feelings already known to women, bringing new meanings to previously known experiences. On the other hand, other strategies to deal with this process begin to be developed by them. Thus, after abandoning the violent cycle, they tend to give new meaning to family relationships: the position occupied by the maternal figure, for example, has a new meaning, understanding that, in fact, their mothers were victims not only of violence, present in different phases of their life, but of the circumstances that were established in their existence, related and interdependent to culture, context and social, economic and family condition, among several other determining contexts for coping with DV.

*We can only give what we have, if my mother didn’t have anything to give, it’s because she hadn’t received it either. I began to understand that her childhood was much more difficult* (...). *She didn’t change, it was me who changed, my gaze on her, I started to look at her as a human being who went through a lot of bad things in life and who didn’t have the chance to understand, to look at herself. Today I understand her reasons.* (Fátima, 32 years old, GA4)

*I understood what happened to my mother, she never had love from anyone, so she couldn’t give me what she didn’t have. I have no regrets, on the contrary, I think she was a great woman within her means, she was a winner.* (Patricia, 44 years old, GA4)

Reframing family relationships implies establishing bonds that did not previously exist. Some women, upon realizing their daughters’ suffering, seek to rebuild relationships through closer dialogue, externalizing feelings of regret, sadness, or impotence given the impossibility of defending the family.

*I never talked to him about anything. Before this happened, we never* (pause)*, it was hi. It wasn’t that deep, family conversation. Then we started to be closer*. (Veronice, 43 years old, GA2)

*When he* (father) *saw me all purple, he felt very bad about everything he did to other people’s daughter. He realized what he did all his life.* (Gabrielle, 32 years old, GA1)

*My father still carries this pain of not being able to defend me* (from violence)*. He found himself powerless over it*. (Elenice, 54 years old, GA1)

In the face of so many painful experiences, the family’s support was recognized after they left the cycle of violence, representing a support network required since childhood. However, for these women, even if unconsciously, the family influenced their insertion in this context of violence, due to the process of subduing, naturalizing and being complacent with the abusive relationships that led, among so many harms, to loss of autonomy, security, self-esteem and independence in relationships.

*I didn’t have my family and, to my surprise, today she is the one who takes care of me. My mom is always supporting me! This* (the violence) *ended up uniting my family.* (Fátima, 32 years old, GA4)

*This negative experience I lived was my family’s influence, I’m sure of that. And I think it also had an influence on getting out of this condition. It seems like it’s a cycle, I don’t know the meaning of it, I can’t find an answer*. (Mariana, 42 years old, GA4)

*I think that unconsciously, because we have witnessed that situation, we end up being attracted to this type of relationship.* (Ilair, 33 years old, GA1)

After the abandonment of DV, new meanings were attributed to family relationships, based on the understanding that family members reproduce a cycle that was experienced over time, elaborating meanings and behaviors in the different situations that arise.

## DISCUSSION

In the context of DV, each situation has a meaning and a consequence, according to the experiences and interaction the woman builds and determines for herself and her relationships. These meanings are attributed individually and even though the experience of many women is similar, the way each one elaborates this context is unique. Thus, throughout a trajectory covered in different contexts of violence, they signify and give new meaning to their family relationships, based on their experiences.

For an individual, the meaning of a given element generally comes from the way other people act towards them in relation to this element. Within this context, SI considers meanings as social products, creations elaborated in and through human activities that determine their interactive process^(9)^. The process, in this study, feeds back on itself: women were born, grown, and developed in a DV context, built by pre-established relationships, which were learned, absorbed, and reproduced by previous generations. This context, at the same time as it is constructed, facilitates the elaboration of new relationships and attributions of meanings that depend on the relationships established at a given moment.

In the process of unwittingly retransmitting violence, women rewrite not only their individual and family history, but the collective history of those who have also found themselves in situations of violence^(13)^. In this study, this finding is demonstrated when women refer to the situation of violence experienced by their mothers, and that these mothers, considered submissive, not loving and/or not protective, did not know how to deal with the violence that ended up being replicated in their lives, and thus get used to living with it. From then on, they perpetuated these patterns as if they were natural and the daughters became victims in their relationships, once again continuing the characteristic family scenario of violence^(13,14)^.

The context experienced revealed gender violence^(15)^, commonly practiced by the male figure – the father, uncle, stepfather, or partner – which refers to a society based on patriarchy and chauvinism present in relationships^(15)^. However, the participants also mentioned the existence of conflicting relationships with their mothers. Similarly, a study describes that men and women perpetrated violence and/or were victimized under the influence of different family experiences, confirming the notion that these behaviors, even when resignified, can be repeated for generations, corroborating the transgenerational perspective of violence^(16)^.

Based on this context, it can be observed that there is a certain difficulty in identifying DV, which may be related to the common sense conception, result of the social and cultural construction, that men is superior to the detriment of women, who are still understood as their submissive^(17)^. In this regard, a study carried out with men in legal process revealed that, in the family context, they believe that women and children must submit to male authority and that men have the right to control them. These experiences, according to them, are absorbed in childhood, when they grasp the meanings and consequently, reproduce similar behaviors as adults^(18)^.

Considering that symbolic interactionism understands meanings as social products, creations elaborated in and through human activities that determine their interactive process^(9)^, it is observed that both male and female figures reinforce and reproduce socially and culturally constructed roles, accepted and naturalized throughout their lives. Thus, experiencing, as a victim or witness, episodes of family violence in childhood offers the subject a model that can be perpetuated, replicating the situation as an adult^(18)^.

A new type of action never originates separately from past experiences^(9)^. The process of coping with and abandoning the cycle of DV for the women in this study showed a lonely path. They understood that they would need, in the first instance, their individual efforts, their available emotional, social, and material resources. After abandoning the DV scenario, the family begins to occupy the space of support, despite the fact that, for many women, the family was the main responsible for insertion in the context, especially due to the fragility in the relationships established with family models. Participants involved in the formation of new joint behavior bring the universe of objects to it, the sets of meanings, and the systematizations of interpretation that they already have. Thus, the new form of joint action, in this case, the women’s attempt to abandon the cycle of DV, always arises from a context of previous collective behaviors, and is associated with it^(9)^.

The family, as the first laboratory of interpersonal relationships, plays roles such as education, socialization experience, development of trust, as well as care and protection^(19)^. Previously, research that sought to analyze social representations of nurses regarding DV against women demonstrated that “family”, for professionals, has both a negative and positive meaning: negative, because violence affects all members, causing adversities in the family nucleus or contributing for intergenerational violence; and positive, as it is in the family that support can be found to break the cycle of DV^(20)^.

It is then understood that one of the strategies for intervening in family relationships based on violence is to modify the interpretation of symbols and encourage the construction of new meanings. In this context, it is worth highlighting the importance of Primary Care, which can seek, through health education actions, to enable the construction of family symbols based on affection, respect, and symmetry between genders^(19)^. Furthermore, with regard to working with children and adolescents, the inclusion of the theme of violence in the School Health Program (PSE), adhered to by municipalities, is an important instrument in this process, as it can cover different realities, socioeconomic levels, microcultures, and age groups^(21)^.

Given the set of narratives in this study, it is inferred that the transformation of the reality of violence extends beyond the health area, that is, it has deep roots and permeates time, generations, cultures, relationships, among other paradigms. Furthermore, it requires the involvement of different areas of activity, such as health, education, social assistance, in actions that seek, mainly, to change the processes in which objects and relationships are created, confirmed, and transformed^(9)^, to favor the identification of children in situations of family abuse, promote relationships of respect and equality between genders, encourage both financial and emotional independence of women who are victims and non-victims of violence. Also noteworthy are remote support services and online support groups, which allowed this study and are configured not only as support, but as an important communication tool, as they provide, through the generation of identification and empathy, the strengthening of women to face violence, in addition to preventing a possible reinsertion into the cycle^(5)^.

### Study Limitations

The limitations of the study in question are related to the difficulty of accessing this audience, especially during the data collection period when the COVID-19 pandemic was taking place, and also due to the difficulty of addressing a sensitive topic. The study was carried out with a population with access to social media, and it is not possible to state that similar results may occur in groups at different levels of accessibility to the *Internet* and social media.

### Contributions to the Area of Nursing/Health

Conversely, the results have the potential to give visibility to the set of meanings of the narratives and the particularities of DV, based on women’s view, from a perspective that goes beyond the focus on violence itself, covering other social actors involved in the context of DV. In this regard, the literature highlights the need for more qualitative studies using technological strategies, considering the importance of continuing research during the pandemic^(22,23)^.

## CONCLUSION

The data analyzed demonstrated the striking stereotype that runs through generations: attribution of strength, dominance and control to the male figure, while the female figure owes obedience, submission, and acceptance. The violence suffered by mothers also affected their children, whether as spectators or victims. Based on their marital choices, women relived situations of violence witnessed in their families, even with the desire to build relationships based on different premises.

The meanings attributed to family relationships are elaborated and modified according to the interpretation, trajectory of confrontation, and interactions of women with other individuals and objects in the social web, over time. They understood that the family had an influence on the insertion in a DV context, due to the relationships permeated by abandonment, lack of protection, submission and dependence, experienced by them from a very early age. By managing to move away from the context of DV, the meanings were reconstructed and, despite the pain, the women began to see family relationships with empathy, understanding the intergenerational experiences established in different contexts, which translated, in many moments, into the reproduction of violence.
